# Radioactive Seed Versus Wire-Guided Localization for Ductal Carcinoma in Situ of the Breast: Comparable Resection Margins

**DOI:** 10.1245/s10434-020-08744-8

**Published:** 2020-06-23

**Authors:** Marie Colombe Agahozo, Sofie A.M. Berghuis, Esther van den Broek, Linetta B. Koppert, Inge-Marie Obdeijn, Carolien H. M. van Deurzen

**Affiliations:** 1 Department of Pathology, Erasmus MC Cancer Institute Rotterdam, Rotterdam, The Netherlands; 2 Department of Health Technology and Services Research, University of Twente, Enschede, The Netherlands; 3 PALGA, Houten, The Netherlands; 4 Department of Surgery, Erasmus MC Cancer Institute, Rotterdam, The Netherlands; 5 Department of Radiology, Erasmus MC Cancer Institute, Rotterdam, The Netherlands

## Abstract

**Background:**

There are currently two widely used methods for preoperative localization of ductal carcinoma in situ (DCIS) of the breast: wire-guided localization (WGL) and radioactive seed localization (RSL). Several studies compared these localization techniques in small cohorts.

**Objective:**

The aim of this study was to compare the surgical resection margin status between RSL and WGL in a large national cohort of patients with DCIS.

**Patients and Methods:**

We included patients from the Dutch Pathology Registry who underwent breast-conserving surgery for DCIS by either RSL (*n* = 1851) or WGL (*n* = 2187) between 2009 and 2019. Several clinicopathological characteristics were compared between these two groups, including resection margin status and number of re-excisions.

**Results:**

Patients undergoing RSL were younger (*p* = 0.014) and were more often diagnosed with a large DCIS (*p* = 0.013), high grade DCIS (*p* < 0.001) and comedonecrosis (*p* < 0.001) compared with patients undergoing WGL. There was no significant difference in resection margin status between both groups (*p* = 0.089) and the number of re-excisions (*p* = 0.429). However, in case of re-excision, patients in the RSL group were more often treated with breast-conserving surgery (*p* = 0.029).

**Conclusion:**

In this large national cohort study of patients with DCIS, we demonstrated that there was no difference in resection margin status between both procedures, or in the number of re-excisions, but patients in the RSL group were more often treated with breast-conserving therapy in case of a re-excision.

Since the introduction and improvement of breast screening by mammography, the detection rate of ductal carcinoma in situ (DCIS) of the breast has increased.^1^^–^3 Nowadays, DCIS accounts for approximately 20% of all breast cancers in the screened population.^1^ The majority of patients with DCIS are treated with surgery, either breast-conserving surgery in combination with radiotherapy, or a mastectomy, depending on the DCIS size and the patients’ preference.^3^^–^5 Clinically, DCIS is generally non-palpable and has a diffuse growth pattern, which makes it challenging to estimate the correct size using imaging modalities.

In literature, there has been a lack of consensus regarding the most optimal resection margin for patients with DCIS. Several authors used different definitions ranging from ‘no ink on the tumor’ to a 10 mm free margin.^6^^,^^7^ Obviously, extensively positive resection margins are associated with a higher risk for local recurrence, but wide margins (> 10 mm) are more likely to compromise cosmetics, therefore this has to be balanced. In 2016, the Society of Surgical Oncology, the American Society for Radiation Oncology, and the American Society of Clinical Oncology published a consensus guideline for patients with DCIS undergoing breast-conserving surgery with whole-breast irradiation. In this guideline, a margin ≥ 2 mm is proposed as being most optimal for patients with DCIS.^8^^,^^9^

In line with international data, there has been a lack of consensus in The Netherlands regarding the optimal resection margin for DCIS. In The Netherlands, resection margin status for patients with invasive breast cancer is defined as free (no ink on tumor), focally positive (≤ 4 mm ink on tumor), or more than focally positive (> 4 mm ink on tumor).^10^^,^^11^ According to this guideline, invasive breast cancer patients with a focally positive margin undergo a boost of radiation instead of re-excision based on the low local recurrence rate in this group.^12^ A re-excision is advised in patients with more than focally positive margins based on the increased local recurrence risk. For patients with DCIS, a re-excision was previously advised for patients with positive margins (ink on tumor), although regional differences were present.^11^ Currently, the Dutch treatment guideline recommends to consider a re-excision for DCIS with a margin < 2 mm, which is consistent with international recommendations.^13^

In order to achieve optimal resection margins at first surgery, proper preoperative DCIS localization is crucial. There are two widely used techniques for DCIS localization: wire-guided localization (WGL) and radioactive seed localization (RSL).^5^ In the case of a WGL procedure, single or multiple wire hooks are placed into the lesion and used as a guide to the lesion,^14^ while in the case of RSL, single or multiple radioactive iodine-I25 seeds are used to mark the lesion.^15^ Although WGL was historically seen as the gold standard, it has several disadvantages.^14^^–^16 The timing of this procedure is dependent on the availability of the radiologist and the surgeon, since the wire hook has to be placed just before surgery. In addition, it causes more discomfort for the patient and may dislocate before or during surgery.^14^^,^^16^ RSL is therefore seen as an attractive alternative;^15^^,^ 16 it allows more flexibility scheduling the surgery, since it can be placed during a longer period before surgery, and causes less patient discomfort.^14^^,^^15^ However, misplaced seeds can only be surgically removed, and, due to its radioactivity, the introduction of RSL in an institution is a logistical challenge since it requires strict regulation and training of all staff involved.^15^

In recent years, several studies have compared both localization techniques but the derived results are inconsistent.^14^^,^^16^^–^18 On the one hand, studies have found comparable results between RSL and WGL in terms of excision margins, resection weight, and the number of re-excisions,^16^^,^^17^ while on the other hand, a lower rate of positive margins and reoperations have been reported for RSL.^14^^,^^18^ However, these studies included small cohorts, usually from one institute, and were not able to demonstrate highly significant differences. In order to present more conclusive results, a large multicenter study including high numbers of patients is required. The aim of this study was to perform a nationwide study, using real-world data, in order to compare the efficacy of WGL versus RSL for patients treated with breast-conserving surgery for DCIS.

## Patients and Methods

### Data Collection and Patient Cohort

All data were obtained from the Dutch Pathology Registry (PALGA), which is the nationwide registry of pathology reports. Since 2009, Dutch pathologists can use synoptic reporting modules to report the pathological test results for several common tumor types, including breast cancer.^19^ In these modules, parameters are captured in numerous standardized variables instead of free-text fields, which facilitates the analyses of all reports simultaneously.

In this study, synoptic reports of all patients diagnosed with primary DCIS who underwent breast-conserving surgery by either RSL or WSL between 2009 and 2019 in The Netherlands were included. Patients with an invasive component in the initial surgical specimen were excluded from further analysis. Data collection included age at diagnosis, several DCIS characteristics (size, grade, presence of comedonecrosis and microcalcifications), type of surgery, resection margins, and number of re-excisions. If available, the estrogen receptor (ER), progesterone receptor (PR), and human epidermal growth factor receptor 2 (HER2) status were also included.

### Statistical Analysis

Variables that were described in the data collection were analyzed using the ‘tableone’ package in R (version 3.5.1; The R Foundation for Statistical Computing, Vienna, Austria). To evaluate potential differences between RSL and WGL, statistical analyses were performed; to evaluate whether categorical variables were significantly different between RSL and WGL, or between the different numbers of seeds injected in the RSL group, the Chi-square test was performed; and to evaluate whether the means of the numerical variables were significantly different, analysis of variance (ANOVA) tests were performed. *P*-values < 0.05 were considered to be significantly different.

## Results

### Baseline Characteristics

A total of 4038 DCIS patients were included, of whom 2187 were treated by WGL and 1851 were treated by RSL. Overall, the mean age was 60.0 years (standard deviation [SD] 9.25). The mean DCIS size was 1.78 cm (SD 1.45) and the majority of DCIS cases were either intermediate or high grade, accounting for 40.8% and 44.6% of cases, respectively. ER, PR, and HER2 status was not available for the majority of patients. Baseline patient and tumor characteristics of the WGL and RSL groups are described and compared in Table 1. Compared with patients treated with WGL, patients with RSL were slightly younger at the time of treatment (59.6 years vs. 60.3 years; *p* = 0.014). Regarding the histopathological aspects of DCIS, patients treated with RSL were more often diagnosed with a larger DCIS size (mean diameter 1.84 cm vs. 1.72 cm; *p* = 0.013), grade 3 DCIS (47.8% vs. 42.0%; *p* < 0.001), and presence of comedonecrosis (92.1% vs. 79.9%; *p* < 0.001) compared with the WGL group.

**Table 1:** Baseline patient and tumor characteristics [*n* = 4038]

	RSL	WGL	*p* value
	[*n* = 1851]	[2187]	
Age, years [mean (SD)]	59.63 (9.48)	60.35 (9.03)	**0.014**
DCIS size, cm [mean (SD)]	1.84 (1.52)	1.72 (1.38)	**0.013**
No. of seeds			
1	920 (49.7)		
2	101 (5.5)		
3	10 (0.5)		
Unknown^a^	820 (44.3)	2187 (100)	
DCIS grade			
1	252 (13.6)	295 (13.5)	**< 0.001**
2	698 (37.7)	951 (43.5)	
3	885 (47.8)	919 (42.0)	
Unknown^a^	16 (0.9)	22 (1.0)	
Comedonecrosis			
Absent	68 (3.7)	252 (11.5)	**< 0.001**
Present	799 (43.2)	1002 (45.8)	
Unknown^a^	984 (53.2)	933 (42.7)	
Microcalcification			
Absent	255 (13.8)	302 (13.8)	0.214
Present	1012 (54.7)	1354 (61.9)	
Unknown^a^	584 (31.6)	531 (24.3)	
Estrogen receptor			
Negative	18 (1.0)	15 (0.7)	0.356
Positive	37 (2.0)	49 (2.2)	
Unknown^a^	1796 (97.0)	2123 (97.1)	
Progesteron receptor			
Negative	26 (1.4)	23 (1.1)	0.292
Positive	22 (1.2)	32 (1.5)	
Unknown^a^	1803 (97.4)	2132 (97.5)	
HER2 status			
Not amplified	3 (0.2)	1 (< 0.1)	1
Amplified	5 (0.3)	2 (0.1)	
Unknown^a^	1843 (99.6)	2184 (99.9)	

Data are expressed as *n* (%) unless otherwise specified

*RSL* radioactive seed localization, *WGL* wire-guided localization, *SD* standard deviation, *DCIS* ductal carcinoma in situ, *HER2* human epidermal growth factor receptor

^a^Excluded from the analysis

Bolded *p*-values indicate significance

### Wire-Guided Localization and Radioactive Seed Localization Have Comparable Surgical Resection Margins

The resection margins are described in Table 2. Overall, around half of the patients had a surgical resection margin ≥ 2 mm, which was not statistically different between the WGL and RSL groups (*p* = 0.505). In line with this, there was no significant difference in the number of re-excisions between both groups (*p* = 0.429). In the WGL group, 13.6% of patients underwent a second surgery and 8.8% underwent a third surgery. In the RSL group, 12.6% of patients underwent a second surgery and 7.7% underwent a third surgery. However, patients with a re-excision after RSL more often underwent a breast-conserving surgery compared with the WGL group (69.2% vs. 59.7%; *p* = 0.029). This resulted in the detection of additional DCIS in 62.8% of patients in both groups. In 1.7% of patients in the WGL group and 2.6% of patients in the RSL group, an invasive component was present in the re-excision material.

**Table 2:** Surgical resection margin status and additional surgery [*n* = 4038]

	RSL [*n* = 1851 (45.8%)]	WGL [*n* = 2187 (54.6%)]	*p* value
Resection margins, cm [mean (SD)]	0.33 (0.29)	0.35 (0.30)	0.089
Resection_margins_cat			
< 2 mm	984 (53.2)	1142 (52.2)	0.505
≥ 2 mm	856 (46.2)	1038 (47.5)	
Unknown^a^	11 (0.6)	7 (0.3)	
Radicality			
Free	1451 (78.4)	1699 (77.7)	0.531
Focally not radical	253 (13.7)	314 (14.4)	
More than focally not radical	136 (7.3)	167 (7.6)	
Unknown^a^	11 (0.6)	7 (0.3)	
First re-excision			
Yes	234 (12.6)	296 (13.5)	0.429
No	1617 (87.4)	1891 (86.5)	
Type of first re-excision			
Breast-conserving surgery	162 (69.2)	176 (59.5)	**0.029**
Mastectomy	72 (30.8)	119 (40.2)	
Unknown^a^	0	1 (0.3)	
Findings in first re-excision specimen			
No rest	80 (34.2)	103 (34.8)	0.888
DCIS	147 (62.8)	186 (62.8)	
LCIS	1 (0.4)	2 (0.7)	
Invasive carcinoma	6 (2.6)	5 (1.7)	
Second re-excision			
Yes	18 (7.7)	26 (8.8)	0.769
No	216 (92.3)	270 (91.2)	
Type of second re-excision			
Breast-conserving surgery	2 (11.1)	2 (7.7)	1
Mastectomy	16 (88.9)	24 (92.3)	
Findings in second re-excision specimen			
DCIS	17 (94.4)	26 (100.0)	0.852
No rest	1 (5.6)	0 (0.0)	

Data are expressed as *n* (%) unless otherwise specified

*RSL* radioactive seed localization, *WGL* wire-guided localization, *SD* standard deviation, *DCIS* ductal carcinoma in situ, *LCIS* lobular carcinoma in situ

^a^Excluded from the analysis

Bolded *p*-values indicate significance

### Number of Seeds and Clinicopathological Characteristics

The association between the number of seeds and clinicopathological characteristics is depicted in Table 3. Overall, the majority of cases (89.2%) were localized by one seed. There was a significant association between the number of seeds and age at diagnosis; a single seed was associated with older age at diagnosis (*p* = 0.017). In addition, single seed localization was associated with a smaller DCIS diameter (*p* < 0.001) and the highest percentage of patients with a resection margin ≥ 2.0 mm (*p* = 0.001) compared with multiple seed localization. However, there was no significant difference in the number of patients undergoing a re-excision (*p* = 0.161).

**Table 3:** Clinicopathological characteristics according to the number of seeds [*n* = 1031]^a^

	Single seed [*n* = 920 (89.2%)]	Multiple seeds (two or three) [*n* = 111 (10.8%)]	*p* value
Age, years [mean (SD)]	59.44 (9.33)	57.16 (10.39)	**0.017**
DCIS size, cm [mean (SD)]	1.70 (1.37)	3.03 (2.14)	**< 0.001**
Resection margins, cm [mean (SD)]	0.36 (0.30)	0.25 (0.24)	**0.002**
Resection_margins_cat			
< 2 mm	448 (48.7)	73 (65.8)	**0.001**
≥ 2 mm	469 (51.0)	37 (33.3)	
Unknown	3 (0.3)	1 (0.1)	
Radicality			
Free	731 (79.5)	88 (79.3)	0.574
Focally not radical	123 (13.4)	12 (10.8)	
More than focally not radical	63 (6.8)	10 (9.0)	
Unknown^b^	3 (0.3)	1 (0.9)	
First re-excision			
Yes	110 (12.0)	19 (17.1)	0.161
No	810 (88.0)	92 (82.9)	

Data are expressed as *n* (%) unless otherwise specified

*SD* standard deviation, *DCIS* ductal carcinoma in situ

^a^Only patients with a known number of seeds were included

^b^Excluded from the analysis

Bolded *p*-values indicate significance

Subgroup analysis was performed by comparing the use of one seed versus more than one seed in patients with a large DCIS (> 3 cm) [Table 4]. The use of multiple seeds was associated with a larger DCIS diameter (*p* = 0.002) and higher rate of radicality (free versus focally irradical versus more than focally irradical; *p* = 0.025). However, there was no association between the number of seeds and the categorical resection margins (< 2 mm vs. ≥ 2; *p* = 1)

**Table 4:** Clinicopathological characteristics of patients with large DCIS according to the number of seeds [*n* = 210]^a^

	Single seed [*n* = 156 (74.3%)]	Multiple seeds (two or three) [54 (25.7%)]	*p* value
Age, years [mean (SD)]	60.17 (9.60)	58.07 (10.71)	0.18
DCIS size [mean (SD)]	4.05 (1.24)	4.73 (1.62)	**0.002**
Resection margins [mean (SD)]	0.19 (0.20)	0.17 (0.17)	0.561
Resection margin categories			
< 2 mm	130 (83.3)	44 (81.5)	1
≥ 2 mm	25 (29.1)	9 (22.0)	
Unknown^b^	1 (0.6)	1 (1.9)	
Radicality			
Free	86 (55.1)	41 (75.9)	**0.025**
Focally not radical	41 (26.3)	5 (9.3)	
More than focally not radical	28 (17.9)	7 (13.0)	
Unknown^b^	1 (0.6)	1 (1.9)	
First re-excision			
Yes	44 (28.2)	11 (20.4)	0.343
No	112 (71.8)	43 (79.6)	

Data are expressed as *n* (%) unless otherwise specified

*DCIS* ductal carcinoma in situ, *SD* standard deviation

^a^Only patients with large DCIS (> 3 cm) with a known number of seeds were included

^b^Excluded from the analysis

Bolded *p*-values indicate significance

## Discussion

The WGL method has historically been used as the gold standard for preoperative localization of non-palpable breast tumors; however, in recent years, RSL has been considered to be an attractive alternative for WGL since it offers more flexibility in scheduling the surgery and is more patient-friendly.^15^ Several, generally small, previous studies compared both methods regarding their surgical outcome, but the derived results were non-consistent.^14^^,^^16^^–^18 In this large, population-based cohort study we compared the efficacy of WGL and RSL in patients with DCIS undergoing breast-conserving surgery.

This study demonstrated that preoperative localization with RSL was more frequently performed in patients who were younger, were diagnosed with a large or high-grade DCIS, or had a DCIS with comedonecrosis. We hypothesize that these differences may be related to an unequal use of these techniques within different hospitals, since the localization technique is an institutional policy and not a patient-dependent choice. Academic hospitals and specialized cancer centers are potentially more likely to use RSL compared with general hospitals, and these centers attract younger patients, whom generally have a higher rate of high-grade lesions compared with older patients. However, the clinical relevance of these differences, which are relatively limited but statistically significant in this large series, is unclear.

When comparing both methods, there was no difference in the surgical resection margins or the number of re-excisions. This is in line with the majority of the studies, which demonstrated equally successful excision rates between RSL and WGL.^14^^,^^16^^,^^17^ However, one previous study suggested significant superiority of RSL over WGL based on 169 patients (*p* = 0.048).^18^ In our study, we observed that in case of a re-excision, patients in the RSL group were more often treated with breast-conserving surgery compared with the WGL group. Potential explanations for this difference could be that these patients are slightly younger. Another hypothesis is that the volume of the initial surgical specimen in patients treated with RSL could be smaller compared with WGL, due to a more accurate localization.

Lastly, we also showed that in terms of RSL, the use of a single seed is associated with a smaller DCIS diameter, which consequently results in a larger resection margin. In our subgroup analysis restricted to patients with extensive DCIS (≥ 3 cm), we demonstrated that the use of multiple seeds was associated with a higher rate of radicality. This is in line with a recent study that also suggested superiority of multiple seeds over a single seed in patients with extensive DCIS (lesions ≥ 3 cm).^20^

Overall, our study included a large, national cohort of patients receiving breast-conserving surgery for DCIS with either WGL or RSL, using real-world data. However, it also has several limitations. Our data used for this analysis, did not contain information regarding the weight or size of the excision specimen. Removal of large excision specimens obviously decreases the rate of positive margins.^14^ The weight of the specimen would provide more information regarding the interpretation of the resection margins and postoperative cosmetics. Additionally, data regarding the method of marker placement (ultrasound-guided or guided by mammography), the number of wires, and the use of intraoperative examination of the specimen and oncoplastic surgery were also lacking, which could have influenced the results. Furthermore, the location of the (involved) margin was not included in the data, which could affect the decision to perform re-excision, i.e. dorsal-involved margins can be less suitable for re-excision, since there might not be any more breast tissue to remove. In addition, we included patients over a period of 10 years, during which the treatment guidelines have changed. Since WGL was used more frequently in the beginning of this period and RSL more frequently towards the end of this period, this could have influenced the re-excision rates. Another limitation is the lack of clinical follow-up data regarding local recurrence, due to the fact that synoptic reporting only began in 2009. Finally, as mentioned above, the localization technique is not a patient-driven choice but an institutional policy, which could have biased our findings.

## Conclusions

Whereas previous studies mainly focused on invasive breast cancer, this study is the first to include a large national cohort using real-world data on patients with DCIS. We have shown that RSL and WGL have comparable resection margins and number of re-excisions. However, in the case of a re-excision, patients in the RSL group were more often treated with breast-conserving surgery compared with the WGL group.
